# SPECTRUM OF DEMENTIA IN OLDER HOSPICE RECIPIENTS

**DOI:** 10.1093/geroni/igac059.1628

**Published:** 2022-12-20

**Authors:** Lauren Hunt, Irena Cenzer, Alexander Smith, Amy Kelley, Melissa Aldridge, Kenneth Covinsky, Krista Harrison

**Affiliations:** University of California, San Francisco, San Francisco, California, United States; University of California, San Francisco, San Francisco, California, United States; University of California, San Francisco, San Francisco, California, United States; Icahn School of Medicine at Mount Sinai, New York, New York, United States; Icahn School of Medicine at Mount Sinai, New York, New York, United States; University of California, San Francisco, San Francisco, California, United States; University of California, San Francisco, San Francisco, California, United States

## Abstract

We know little about differences between hospice enrollees with dementia co-existing with another terminal illness (like cancer), those dying from dementia (as principal hospice diagnosis), and those dying with no dementia. We used the National Health Aging and Trends Study linked to Medicare claims to compare characteristics, hospice use patterns, and care quality ratings. Among 1,105 decedent hospice-enrollees age 70+, we found 39% were dying with coexisting dementia, 17% from dementia, and 44% without dementia. In adjusted analyses, those dying with dementia had similarly high rates of functional impairment, higher rates of clinical needs, and worse measures of care quality compared to elders dying from dementia. Hospice use patterns were different for elders dying with dementia compared to elders without dementia. In summary, 56% of older hospice enrollees have dementia, mostly in addition to another terminal illness. Their differing hospice experience implies changes are needed to hospice care and policy.

